# Facilitated WhatsApp Support Groups for Youth Living With HIV in Nairobi, Kenya: Single-Arm Pilot Intervention Study

**DOI:** 10.2196/49174

**Published:** 2023-11-13

**Authors:** Keshet Ronen, Cyrus Mugo, Anne Kaggiah, David Seeh, Manasi Kumar, Brandon L Guthrie, Megan A Moreno, Grace John-Stewart, Irene Inwani

**Affiliations:** 1 Department of Global Health University of Washington Seattle, WA United States; 2 Department of Epidemiology University of Washington Seattle, WA United States; 3 Department of Research and Programs Kenyatta National Hospital Nairobi Kenya; 4 Department of Psychiatry University of Nairobi Nairobi Kenya; 5 New York University Langone Health New York, NY United States; 6 Department of Pediatrics University of Wisconsin - Madison Madison, WI United States; 7 Department of Medicine University of Washington Seattle, WA United States; 8 Department of Pediatrics University of Washington Seattle, WA United States; 9 Department of Pediatrics Kenyatta National Hospital Nairobi Kenya

**Keywords:** HIV, mHealth, social media, youth, adolescent, social support

## Abstract

**Background:**

Mobile technology can support HIV care, but studies in youth are limited. In 2014, youth receiving HIV care at several health care facilities in Nairobi, Kenya spontaneously formed peer support groups using the social media platform WhatsApp.

**Objective:**

Inspired by youth-initiated groups, we aimed to evaluate the use of WhatsApp to deliver a social support intervention to improve HIV treatment and psychosocial outcomes in youth. We developed a facilitated WhatsApp group intervention (named Vijana-SMART), which was grounded in social support theory and guided by the design recommendations of youth living with HIV. This paper evaluates the intervention’s acceptability and pre-post changes in health outcomes.

**Methods:**

The intervention involved interactive WhatsApp groups facilitated by study staff for 6 months, with each group having approximately 25 members. Study staff sent weekly structured messages, and the message content was based on social support theory and encouraged unstructured peer-to-peer messaging and support. We conducted a single-arm pilot among 55 youth living with HIV aged 14-24 years recruited from a government health care facility serving a mixed-income area of Nairobi. At enrollment and follow-up, self-report questionnaires assessed acceptability; antiretroviral therapy (ART) information, motivation, and behavioral skills (IMB); depression; social support; stigma; resilience; and ART adherence. All participants received the intervention. We used generalized estimating equations (GEEs) clustered by participant to evaluate changes in scores from baseline to follow-up, and correlates of participant WhatsApp messaging.

**Results:**

The median participant age was 18 years, and 67% (37/55) were female. Intervention acceptability was high. All participants reported that it was helpful, and 73% (38/52) sent ≥1 WhatsApp message. Messaging levels varied considerably between participants and were higher during school holidays, earlier in the intervention period, and among youth aged ≥18 years. IMB scores increased from enrollment to follow-up (66.9% to 71.3%; *P*<.001). Stigma scores also increased (8.3% to 16.7%; *P*=.001), and resilience scores decreased (75.0% to 70.0%; *P*<.001). We found no significant change in ART adherence, social support, or depression. We detected a positive association between the level of messaging during the study and the resilience score, but no significant association between messaging and other outcomes. Once enrolled, it was common for participants to change their phone numbers or leave the groups and request to be added back, which may present implementation challenges at a larger scale.

**Conclusions:**

Increased IMB scores following WhatsApp group participation may improve HIV outcomes. Increased stigma and decreased resilience were unintended consequences and may reflect transient effects of group sharing of challenging experiences, which should be addressed in larger randomized evaluations. WhatsApp groups present a promising and acceptable modality to deliver supportive interventions to youth living with HIV beyond the clinic, and further evaluation is warranted.

**Trial Registration:**

ClinicalTrials.gov (NCT05634265); https://clinicaltrials.gov/study/NCT05634265

## Introduction

In 2021, an estimated 1.71 million adolescents aged 10-19 years were living with HIV globally, with 86% of them in sub-Saharan Africa [1]. Youth aged 15-24 years made up 28% of new HIV infections [2]. Advances in pediatric HIV treatment have greatly improved child survival, but have not improved adolescent and youth outcomes to the same extent [2,3]. Youth living with HIV experience poor outcomes at every step of the care cascade: diagnosis, linkage to care, antiretroviral therapy (ART) adherence, retention in care, virologic suppression, and psychosocial health [4-10].

The use of mobile communication technology for health (mobile health [mHealth]) is a promising strategy to improve youth HIV outcomes. Access to mobile phones and social media has grown exponentially over the last decade in both high-income and low-income settings. In Kenya in 2021, of the total population of 54.4 million, there were 59.2 million mobile phone subscriptions (109% coverage) and 20.9 million mobile internet users (38% coverage) [11]. Recent systematic reviews suggest that mHealth can improve ART adherence and retention in care, though individual study findings are mixed [12,13]. Studies to date have mostly employed SMS text messaging to connect individual adults living with HIV with health care workers. Fewer interventions have focused on youth or on using virtual groups through platforms, such as WhatsApp, to connect people living with HIV with each other [14,15]. Peer group interventions may be especially appealing and impactful for youth, whose developmental stage and social position can heighten the impact of peer influences [16].

In 2014, youth receiving HIV care at several health care facilities in Nairobi, Kenya, spontaneously formed peer support groups using the social media platform WhatsApp. Interviews with participants in these youth-led groups indicated that the groups served to supplement in-person support groups and provide information, emotional support, and community to youth living with HIV [17]. Inspired by these groups and guided by input from youth living with HIV, we developed a standardized WhatsApp group intervention to support engagement in HIV care among youth living with HIV. The intervention was developed based on formative interviews with youth living with HIV and was grounded in social support theory [17,18]. Here, we report the uptake, utility, and preliminary effect of the intervention, named Vijana-SMART, on the HIV and mental health outcomes of participants.

## Methods

### Study Design

The study involving Vijana-SMART was a single-arm pilot study with pre-post longitudinal outcome assessment. This design was used in this initial pilot owing to funding and time constraints.

### Study Participants

This study was conducted at a level 3 government health care facility serving a mixed-income area of Nairobi including formal and informal housing, where no other social media peer groups were offered. Youth receiving HIV care were recruited in person by the study team and through paper flyers posted at the facility. Facility staff also shared information about the study and referred potential participants. Snowball sampling from enrolled participants was used to maximize recruitment of youth aged <18 years. Interested participants provided verbal consent for study staff to complete eligibility screening with a tablet-based electronic questionnaire administered using the Open Data Kit. Eligible participants were aged 14-24 years, were living with HIV and aware of their status, and had access to WhatsApp. Participants provided written informed consent prior to enrollment. In accordance with Kenyan regulations, parent or guardian permission was required for adolescents aged 14-17 years, unless they were considered emancipated minors due to being married, pregnant, or parenting. Ethical review boards additionally granted a waiver of parental permission for adolescents who were attending HIV care without a parent or guardian. Participants were enrolled between January 31 and March 26, 2019. Follow-up visits were completed between November 18 and December 14, 2019.

### Intervention

The Vijana-SMART intervention involved a facilitated peer group for youth living with HIV, which was delivered through WhatsApp. The intervention was inspired by spontaneously developed youth-led WhatsApp support groups at other facilities in Nairobi and was designed based on youth guidance in 2 rounds of individual interviews and focus groups with youth living with HIV [17]. Intervention messages were developed based on findings from formative interviews and the social support theory [18], which posits that individuals experience social support through informational, instrumental, companionship, and emotional forms. In keeping with this theory, messages were developed to communicate each domain of support and highlighted key priorities in HIV care, such as destigmatizing oneself, taking care of one’s health, connecting with health workers, and building social ties. Our approach was to adopt an “adolescent responsive” and “adolescent friendly” stance in offering these support groups. Our behavioral strategies therefore were nonjudgmental light-touch messages to strengthen social support, reiterated in order to drive home the value of these simple procedures in everyday life. The presence of a buffering “safe” adult facilitator in the groups was another strategy to modulate prosocial and care responsive behaviors and practices. Peer discussion of topics was encouraged as a core domain of the intervention, with peers having the unique power to amplify content from the messages. In accordance with formative input, participants were split into 2 WhatsApp groups by age (<18 and ≥18 years old), each with approximately 25 members. An in-person launch meeting was conducted at the health care facility for each group to allow participants to meet, name their group, agree on group norms, and choose a day and time to receive their weekly message. A study team member trained in public health and HIV counseling and with 5 years of experience at a hospital youth center then facilitated both groups for 6 months, by sending weekly scheduled messages, answering participant questions, and encouraging group discussion. Intervention messages were designed based on guidance from formative interviews [17], and the topics included ART adherence, medication side effects, nutritional practices, depression, social support, HIV status disclosure, stigma, positive prevention, substance use, and contraception (Multimedia Appendix 1). Messages were the same in both groups, based on input from participants in formative work. All participants received the intervention. Three months of content was developed prior to initiation. At 3 months, the facilitator asked the groups which topics they wanted to explore more, and messages regarding these topics were added in the subsequent weeks. To protect participant confidentiality, study messages avoided the use of explicit HIV-related language. Youth could message the group or individuals in it at any time, and the facilitator responded to questions within 24 hours. Other members of the study team with expertise in HIV clinical care and youth mental health supported the facilitator in responding to challenging questions. The facilitator also corrected misinformation and monitored the conversation for behavior that violated group norms. Group messages were reviewed by the facilitator (DS) and study principal investigator (KR) on a weekly basis for quality control. Message development was a collaborative effort by study investigators, whose expertise included HIV clinical care for youth in Kenya, mental health in youth living with HIV, and the effect of social media on youth behavior.

### Data Collection

Participants attended 2 in-person study visits at the study facility, at enrollment and 6 months. At each study visit, study staff administered self-report tablet-based questionnaires to assess ART information, motivation, and behavioral skills (IMB; using 16 questions from the LifeWindows IMB tool [19]); depression symptoms (using the Patient Health Questionnaire-9 [PHQ-9] [20]); social support (using social support behaviors [SS-B] [21]); stigma (using an abbreviated 6-question version of the HIV-stigma subscale for adolescents living with HIV [ALHIV-SS] [22], which assessed anticipated, experienced, and internalized stigma); resilience (using an abbreviated 5-question version of the Connor-Davidson scale [23]); and ART adherence (using the Wilson 3-item scale [24], which generates a percentage score based on self-assessment of adherence). Participant demographic characteristics and HIV history were also collected at enrollment. At the exit, participants answered a series of questions on the acceptability and utility of Vijana-SMART, and a subset of participants completed an in-depth interview. Qualitative findings have been previously reported [25], and this manuscript focuses on questionnaire data. Group and one-to-one WhatsApp messages were exported weekly. Participants were offered reimbursement of 400 KSh (approximately US $4) as compensation for their travel expenses and time participating in each study visit. The sample size was selected to detect a difference in the mean depression score of 4.1 versus 1.6 using the PHQ-9 and in the ART IMB score of 23 versus 27 using the LifeWindows IMB scale.

### Data Analysis

We assessed individual participant-level changes from baseline to follow-up in the following primary outcomes: ART IMB, ART adherence, depression symptoms, social support, stigma, and resilience. Instrument scores at enrollment and follow-up were modeled by generalized estimating equations (GEEs) clustered by participant. Intent-to-treat analyses were performed, and they were conducted among all participants and among those who had data available at both enrollment and follow-up (complete cases) to explore possible biases due to loss to follow-up. The mode of HIV acquisition was determined based on self-report if the participant knew the mode of acquisition. If none was reported, age at infection or ART initiation was used as a proxy, with the age of ≤14 years considered perinatal. Participant satisfaction with the intervention was summarized descriptively.

Participant engagement in the intervention was assessed as follows: (1) Boolean indicator of whether participants left the group during the intervention, (2) total number of messages sent per day, (3) Boolean indicator of whether the participant was active (ie, sent ≥1 message on a given day). We evaluated the association of baseline characteristics (as predictors) with each measure of engagement (as outcomes). Leaving the group was modeled using Poisson regression, the number of messages sent per day was modeled using linear GEEs clustered by participant, and being active each day was modeled using log-binomial GEEs clustered by participant. We evaluated the association of each measure of engagement (each as a predictor) with each behavioral outcome (IMB score, depression symptoms, ART adherence, social support, and resilience), using linear regression adjusted for the baseline score of the outcome variable. Analyses were conducted using Stata v13 (StataCorp) and RStudio v2022.07.1+554 (Posit Software, PBC).

### Ethics Approval

This research was approved by the ethics review boards at the University of Washington (STUDY00002554) and Kenyatta National Hospital/University of Nairobi (P296/06/2017), and performed in accordance with the ethical standards as laid down in the 1964 Declaration of Helsinki and its later amendments. The study was registered at ClinicalTrials.gov (NCT05634265). Registration was completed after data collection because the study was not determined to require registration by the ethics review boards.

## Results

### Intervention Uptake

**Figure 1:**
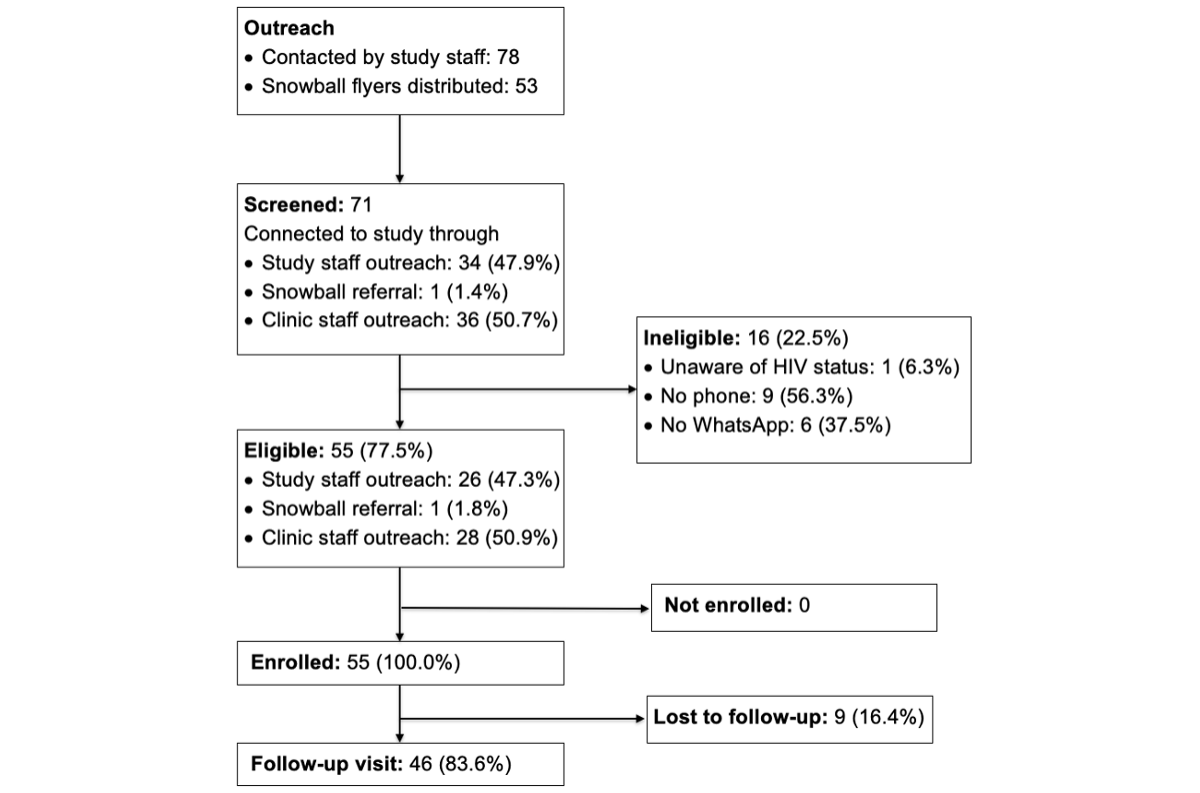
Participant flow. The number of participants is summarized at each stage.

Figure 1 summarizes the flow of study participants. Between January 31, 2019, and March 26, 2019, the study team approached a total of 78 youth attending HIV care at the study clinic. Of these, 71 were screened for eligibility and 55 (78%) were eligible to enroll. Of the 16 ineligible participants, 15 lacked access to a phone or to WhatsApp. All 55 eligible participants were enrolled and assigned to the intervention, and 46 (84%) completed 6-month follow-up visits. Of the 55 participants, 53 were given flyers to recruit youth aged <18 years in their network. Of the forms of recruitment used, outreach by the clinic and study staff accounted for the largest proportions of participants recruited: 47% (26/55) of enrolled participants first learned about the study from study staff, 51% (28/55) learned about the study from clinic staff, and 2% (1/55) learned about the study from another participant through snowball sampling.

### Participant Characteristics

Participant characteristics are summarized in Table 1. The median age was 18 years (IQR 16-22). Among the 55 participants, 37 (67%) were female, most had completed at least primary education (primary: 29/55, 53%; secondary: 13/55, 24%; above secondary: 6/55, 11%), and approximately half (29/55, 53%) were still in school (4/29, 14% were in boarding school). Most participants (39/55, 71%) lived with their parent or guardian, and 20 (39%) had experienced loss of a parent before the age of 18 years. Around half of the participants (29/55, 53%) had acquired HIV perinatally. All participants were on ART, with a median of 4.0 years (IQR 1.0-8.0) since initiation. Around a third (17/55, 31%) were already participating in in-person support groups for youth living with HIV, and 1 participant was in a virtual peer support group. Under half of the participants (22/55, 40%) shared their phone with someone else (with a parent: 8/55, 15%; with a sibling or other relative: 7/55, 13%; with a partner: 5/55, 9%; with a friend: 2/55, 4%). Approximately two-thirds (37/55, 67%) had previously used WhatsApp. The characteristics of the 46 participants who completed follow-up and the 9 who were lost to follow-up were mostly similar, except that more retained participants were currently in school (27/46, 59% vs 2/9, 22%; *P*=.05) and fewer were employed (8/46, 17% vs 6/9, 67%; *P*=.004) (Table 2).

**Table 1:** Participant baseline characteristics.

Characteristic		Value
Age (N=55), median (IQR)		18 (16-22)
Female (N=55), n (%)		37 (67)
**Education level (N=55), n (%)**		
	Primary incomplete	7 (13)
	Primary complete	29 (53)
	Secondary complete	13 (24)
	Above secondary	6 (11)
Currently in school (N=55), n (%)		29 (53)
In boarding school (N=55), n (%)		4 (7)
Living with a parent or guardian (N=55), n (%)		39 (71)
**Orphanhood (N=51), n (%)**		
	None	31 (61)
	Maternal only	2 (4)
	Paternal only	12 (24)
	Both	6 (12)
Employed (N=55), n (%)		13 (24)
**Source of financial support^a^ (N=55), n (%)**		
	Parent or guardian	29 (53)
	Self	11 (20)
	Nonparent relative	10 (18)
	Partner	7 (13)
	Friend	1 (2)
**Mode of HIV acquisition^b^ (N=55), n (%)**		
	Perinatal	29 (53)
	Horizontal	24 (44)
	Unknown	2 (4)
On ART^c^ (N=55), n (%)		55 (100)
Years since ART start (N=47), median (IQR)		4.0 (1.0-8.0)
Usually accompanied on clinic visits (N=55), n (%)		10 (18)
Has people supporting treatment (N=55), n (%)		21 (38)
Status known by anyone (N=55), n (%)		52 (95)
Status disclosed to anyone (N=55), n (%)		31 (56)
**In a peer support group^a^ (N=55), n (%)**		
	In person	17 (31)
	Virtual	1 (2)
Phone shared (N=55), n (%)		22 (40)
**Use of phone communication^a^ (N=55), n (%)**		
	SMS text message	51 (93)
	Phone call	51 (93)
	WhatsApp	34 (62)
Ever used WhatsApp (N=55), n (%)		37 (67)
Airtime cost per week (KSh)^d^ (N=38), median (IQR)		140 (70-250)
Data cost per week (KSh)^d^ (N=31), median (IQR)		100 (50-210)

^a^Not mutually exclusive.

^b^Mode of acquisition reported by the participant, if reported. If no mode of acquisition was reported, age at infection was used as a proxy, with age <14 years coded as perinatal and 14 years coded as horizontal.

^c^ART: antiretroviral therapy.

^d^A currency exchange rate of KSh 100=US $1 is applicable.

**Table 2:** Baseline characteristics of participants who did and did not complete follow-up.

Characteristic			Retained					Lost to follow-up					*P* value		
			N		Value		N			Value					
Age, median (IQR)			46		17 (16-22)		9			20 (19-22)		.23			
Female, n (%)			46		29 (63)		9			8 (89)		.13			
**Education level, n (%)**			46				9					.16			
	Primary incomplete			7 (15)					0 (0)						
	Primary complete			23 (50)					6 (67)						
	Secondary complete			12 (26)					1 (11)						
	Above secondary			4 (9)					2 (22)						
Currently in school, n (%)			46		27 (59)		9			2 (22)		.05			
In boarding school, n (%)			46		4 (9)		9			0 (0)		.36			
Living with a parent or guardian, n (%)			46		33 (72)		9			6 (67)		.76			
**Orphanhood, n (%)**			43				8					.72			
	None			27 (63)					4 (50)						
	Maternal only			2 (5)					0 (0)						
	Paternal only			9 (21)					3 (38)						
	Both			5 (12)					1 (13)						
Employed, n (%)			46		8 (17)		9			6 (66)		.004			
**Source of financial support^a^, n (%)**			46				9								
	Parent or guardian			26 (57)					3 (33)		.20				
	Self			8 (17)					3 (33)		.27				
	Nonparent relative			5 (11)					0 (0)		.30				
	Partner			6 (13)					1 (11)		.87				
	Friend			1 (2)					0 (0)		.66				
**Mode of HIV acquisition^b^, n (%)**			46				9					.16			
	Perinatal			15 (33)					5 (56)						
	Horizontal			18 (39)					4 (44)						
	Unknown			13 (28)					0 (0)						
On ART^c^, n (%)			46		46 (100)		9			9 (100)		>.99			
Years since ART start, median (IQR)			40		3.5 (1-8)		7			5 (1-7)		.89			
Usually accompanied on clinic visits, n (%)			46		8 (17)		9			2 (22)		.73			
Has people supporting treatment, n (%)			46		17 (37)		9			4 (44)		.67			
Status known by anyone, n (%)			46		44 (96)		9			8 (89)		.41			
Status disclosed to anyone, n (%)			46		25 (54)		9			6 (67)		.50			
**In a peer support group^a^, n (%)**			46				9								
	In person			16 (35)					1 (11)		.16				
	Virtual			0 (0)					1 (11)		.02				
Phone shared, n (%)			46		20 (44)		9			2 (22)		.23			
**Use of phone communication^a^, n (%)**			46				9								
	SMS text message			42 (91)					9 (100)		.36				
	Phone call			42 (91)					9 (100)		.36				
	WhatsApp			28 (61)					6 (67)		.74				
Ever used WhatsApp, n (%)			46		60 (65)		9			7 (78)		.46			
Airtime cost per week (KSh)^d^, median (IQR)			60		140 (70-250)		8			125 (60-350)		.20			
Data cost per week (KSh)^d^, median (IQR)			24		100 (60-200)		7			70 (20-210)		.58			

^a^Not mutually exclusive.

^b^Mode of acquisition reported by the participant, if reported. If no mode of acquisition was reported, age at infection was used as a proxy, with age <14 years coded as perinatal and 14 years coded as horizontal.

^c^ART: antiretroviral therapy.

^d^A currency exchange rate of KSh 100=US $1 is applicable.

### Intervention Acceptability and Use

Intervention acceptability was very high among the 46 participants who completed follow-up (Table 3). Among those who responded, 100% (40/40) strongly or somewhat agreed that Vijana-SMART was helpful overall, 100% (42/42) would recommend it to a friend, 100% (40/40) reported that they obtained new information, 95% (38/40) reported that it helped them adhere to ART, and 98% (39/40) reported that it helped them emotionally. A minority of the participants (7/41, 17%) reported discussing message content with friends outside the group. Few participants reported that they avoided an unnecessary clinic visit (2/39, 5%) or sought clinic-based care (6/41, 15%) because of advice or information they received through Vijana-SMART.

**Figure 2:**
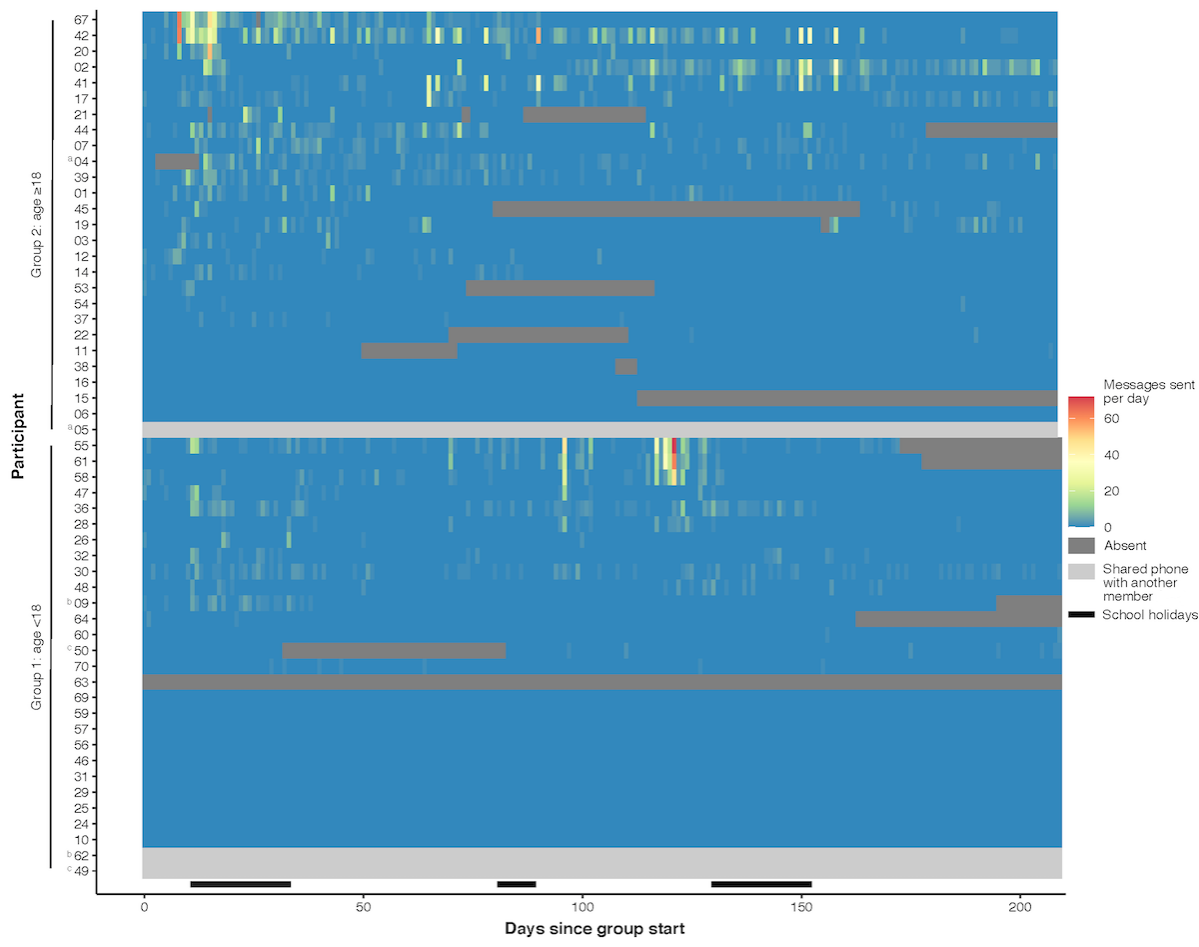
Participant messaging over time in each of the intervention groups. Each tile represents a day in a participant’s messaging, colored by the number of messages the participant sent during that day. Dark grey represents periods when the participant left the group. Light grey represents participants who were in the group but were connected through another member’s phone, who was a member of the household. Participants 4 and 5 used the phone of participant 4; participants 9 and 62 used the phone of participant 9; and participants 50 and 49 used the phone of participant 50. Participants are sorted by the level of messaging over the intervention period.

As empirical measures of intervention engagement, we examined participant messaging levels in the group. Figure 2 illustrates the daily number of WhatsApp messages sent by each participant over time, and Table 3 presents summary statistics. Among 52 participants, 8 (15%) exited the group early and an additional 10 (19%) temporarily left the group during follow-up (Figure 2). For the latter, when the facilitator reached out to the participants by WhatsApp message or phone call, the participants indicated that they had left in error or due to a change in the phone number and requested to be added back to the group. A further 3 participants shared a WhatsApp account with another member of the group and therefore did not message on their own account. Of the 52 participants whose WhatsApp accounts were used, most participants (38/52, 73%) sent at least 1 message to the group. The median number of messages sent per participant over the 6-month intervention period was 28.5 (IQR 0-105.5), and the median number of days a participant sent any messages was 8.5 (IQR 0-31.5) (Table 3).

**Table 3:** Participant satisfaction with the Vijana-SMART intervention at follow-up.

Variable	Value
Vijana-SMART was overall helpful^a^ (N=40), n (%)	40 (100)
Would recommend Vijana-SMART to a friend^a^ (N=42), n (%)	42 (100)
Vijana-SMART taught new information^a^ (N=40), n (%)	40 (100)
Vijana-SMART helped adhere to ART^a,b^ (N=40), n (%)	38 (95)
Vijana-SMART helped emotionally^a^ (N=40), n (%)	39 (98)
Discussed message content with a friend (N=41), n (%)	7 (17)
Avoided clinic visit due to Vijana-SMART message (N=39), n (%)	2 (5)
Attended clinic visit due to Vijana-SMART message (N=41), n (%)	6 (15)
Left the group during the intervention (N=52), n (%)	18 (35)
Rejoined the group (N=18), n (%)	10 (56)
Sent ≥1 message to the group (N=52), n (%)	38 (73)
Total number of messages sent to the group (N=52), median (IQR)	28.5 (0-105.5)
Number of days any message was sent to the group (N=52), median (IQR)	8.5 (0-31.5)

^a^Somewhat or strongly agree.

^b^ART: antiretroviral therapy.

Messaging patterns varied considerably between participants (Figure 2). We observed clusters of a few days of high daily message counts by multiple participants, indicating a particularly engaged group conversation (eg, participants 67, 42, and 20 in group 2 on days 8-16; participants 42 and 2 in group 2 on days 100-160; and participants 55, 61, and 58 in group 1 on days 117-124). Analysis of correlates of longitudinal engagement indicated that messaging was significantly higher during school holidays and significantly declined over time since the intervention start, and that participants in the ≥18 age group were significantly more likely to send ≥1 daily message than those in the <18 age group (Table 4). We found no significant associations between participant baseline characteristics and the level of messaging or probability of leaving the group. Qualitative analyses of participants’ messaging content and experiences with the intervention have been published elsewhere [25]. Messaging topics in the periods of the highest engagement varied. In group 2 on days 8-16, facilitator messages were related to the in-person launch meeting, while participant messages were related to building social connections, experiences learning their status, disclosure to others, ART side effects, negotiating condom use, and 1 participant’s experience of their baby’s death from HIV. In group 2 on days 100-160, facilitator-scheduled messages were related to stigma, mental health, relationships, substance use, and medical independence, and participant messages were related to stigma, reaching undetectable viral load, participants’ children’s HIV status, disclosure to partners, sharing medication with family members, and side effects. In group 1 on days 117-124, facilitator-scheduled messages were related to mental health and relationships, while participant messages were related to in-person meetings.

When asked, no participants reported experiencing a confidentiality breach in which a third party accessed their Vijana-SMART messages. However, 3 instances of third-party access were reported during implementation or in-depth exit interviews: participant 21 exited the group and, when asked why, explained that their sibling had accessed their phone and exited the group; participant 39’s sibling accessed the group and had an exchange with another group member; and participant 42 reported messaging one-to-one with another participant and then realizing they were messaging with the participant’s spouse who had accessed the phone [25]. All 3 participants continued with the intervention after the event.

**Table 4:** Correlates of participant engagement in the intervention.

Variable^a^				Left group at any time^b^ (overall 18/52^c^, 35%)					Number of messages sent per day^d^ (overall mean 0.41, 95% CI 0.21-0.62)					Any messages sent per day^e^ (overall 1100/10894^f^, 10%)		
				RR^g^ (95% CI)		*P* value		Coefficient (95% CI)			*P* value		RR (95% CI)			*P* value
**Baseline correlates**																
	Age			1.12 (0.96 to 1.29)		.14		0.05 (−0.01 to 0.11)			.12		1.10 (1.00 to 1.22)			.06
	**WhatsApp group**															
		Group 1	Reference				Reference					Reference				
		Group 2	1.85 (0.70 to 4.93)		.22		0.36 (−0.03 to 0.75)			.07		2.17 (1.08 to 4.35)			.03	
	Female			2.65 (0.77 to 9.14)		.12		0.10 (−0.33 to 0.52)			.66		0.76 (0.38 to 1.51)			.44
	**Education level**															
		Primary incomplete	Reference				Reference					Reference				
		Primary complete	1.04 (0.22 to 4.88)		.96		0.17 (−0.40 to 0.75)			.56		0.99 (0.23 to 3.09)			.99	
		Secondary complete	1.75 (0.35 to 8.67)		.49		0.73 (0.09 to 1.38)			.03		2.26 (0.74 to 6.93)			.15	
		Above secondary	1.17 (0.16 to 8.28)		.88		0.24 (−0.52 to 1.00)			.53		1.42 (0.37 to 5.49)			.61	
	Currently in school			0.46 (0.17 to 1.23)		.12		−0.26 (−0.65 to 0.14)			.21		0.64 (0.33 to 1.26)			.20
	Years since ART^h^ start			0.95 (0.84 to 1.07)		.39		−0.03 (−0.09 to 0.02)			.20		0.95 (0.87 to 1.04)			.29
	Status known by anyone			1.04 (0.14 to 7.82)		.97		−0.45 (−1.30 to 0.41)			.31		0.50 (0.18 to 1.37)			.18
	Status disclosed to anyone			1.25 (0.48 to 3.21)		.65		0.04 (−0.37 to 0.44)			.86		1.02 (0.51 to 2.03)			.95
	Phone shared			1.39 (0.55 to 3.52)		.49		−0.47 (−0.86 to 0.07)			.02		0.46 (0.20 to 1.04)			.06
	Ever used WhatsApp			1.07 (0.12 to 9.13)		.95		0.08 (−0.49 to 0.65)			.77		0.71 (0.10 to 4.78)			.72
	In an in-person support group			0.64 (0.21 to 1.95)		.44		0.02 (−0.42 to 0.45)			.95		0.86 (0.40 to 1.86)			.70
	Baseline IMB^i^ score			1.01 (0.93 to 1.11)		.77		0.02 (−0.02 to 0.06)			.30		1.02 (0.95 to 1.08)			.64
	Baseline social support			1.00 (0.96 to 1.04)		.94		0.002 (−0.01 to 0.02)			.84		1.03 (1.00 to 1.06)			.06
	Baseline stigma score			1.00 (0.98 to 1.03)		.78		0.002 (−0.0 to 0.01)			.67		1.00 (0.98 to 1.02)			.97
	Baseline ART adherence score			1.02 (0.98 to 1.07)		.35		0.01 (−0.01 to 0.02)			.36		1.02 (0.99 to 1.05)			.20
	Baseline depression score			0.99 (0.87 to 1.13)		.89		0.02 (−0.04 to 0.07)			.56		1.00 (0.91 to 1.10)			.96
**Time-varying correlates**																
	School holiday			N/A^j^				0.22 (0.16 to 0.33)			<.001		1.42 (1.25 to 1.60)			<.001
	Time since intervention start			N/A				−0.003 (−0.003 to −0.002)			<.001		0.99 (0.99 to 1.00)			<.001

^a^Analyses evaluate the association between correlates as predictors and engagement measures as outcomes. All analyses exclude 3 participants who did not message on their own devices but shared phones with 3 other participants.

^b^Cross-sectional Poisson regression estimating the association of baseline characteristics with the risk of ever leaving.

^c^Number of participants who left the group at any time.

^d^Repeated measures generalized estimating equation (GEE) linear regression estimating the association of baseline and time-varying characteristics with the daily number of messages sent.

^e^Repeated measures GEE log-binomial regression estimating the association of baseline and time-varying characteristics with the daily risk of sending ≥1 message.

^f^Number of participant-days on which participants sent ≥1 message.

^g^RR: risk ratio.

^h^ART: antiretroviral therapy.

^i^IMB: information, motivation, and behavioral skills.

^j^N/A: not applicable.

### Pre-Post Change in HIV-Related Outcomes

Table 5 summarizes measures at enrollment and 6-month follow-up visits. In a complete case analysis restricted to participants who had outcome measures at both timepoints, we found that the ART IMB score was significantly higher at follow-up than at enrollment, increasing from a median score of 66.9 (out of 100) to 71.3 at follow-up, an increase of 6.4 (95% CI 3.1-9.6; *P*<.001). Within the IMB score, the information and behavior skills elements both significantly increased at follow-up, with an increase in the score of 6.5 (95% CI 2.2-10.8; *P*=.003) and 6.1 (95% CI 0.6-11.6; *P*=.03), respectively. The self-reported ART adherence score remained similar between enrollment and follow-up (88.9 vs 92.2, out of a maximum of 100; *P*=.50).

**Table 5:** Pre-post comparison of HIV treatment and psychosocial outcomes over the intervention period.

Outcome		Enrollment^a^			Follow-up			Coefficient (95% CI)^b^		*P* value
		N	Value, median (IQR)	N		Value, median (IQR)				
**ART^c^ IMB^d^ score^e^**		38	66.9 (65.0-71.3)	38		71.3 (67.5-78.8)	6.4 (3.1 to 9.6)		<.001	
	ART information	41	73.3 (66.7-80.0)	41		83.3 (73.3-86.7)	6.5 (2.2 to 10.8)		.003	
	ART motivation	43	56.0 (44.0-64.0)	43		60.0 (44.0-68.0)	3.7 (−1.7 to 9.1)		.18	
	ART behavioral skills	44	74.0 (64.0-80.0)	44		80.0 (68.0-88.0)	6.1 (0.6 to 11.6)		.03	
ART adherence^e^		46	88.9 (77.8-94.4)	46		92.2 (77.8-97.8)	2.1 (−3.9 to 8.0)		.50	
Depression symptoms^f^		46	1.0 (0.0-4.0)	46		3.0 (0.0-5.0)	0.8 (−0.6 to 2.1)		.26	
**Stigma score^e^**		39	8.3 (0.0-16.7)	39		16.7 (0.0-33.3)	10.3 (3.7 to 16.6)		.001	
	Anticipated stigma	42	50.0 (0.0-50.0)	42		50.0 (0.0-50.0)	13.1 (−1.7 to 27.9)		.08	
	Enacted stigma	43	0.0 (0.0-0.0)	43		0.0 (0.0-25.0)	4.1 (−1.0 to 9.2)		.12	
	Internalized stigma	43	0.0 (0.0-0.0)	43		16.7 (0.0-33.3)	10.1 (2.3 to 17.9)		.01	
Resilience score^e^		41	75.0 (65.0-85.0)	41		70.0 (55.0-80.0)	−11.7 (−18.2 to −5.2)		<.001	
Social support score^e^		37	59.2 (54.2-71.7)	37		65.8 (52.5-69.2)	1.2 (−5.4 to 7.8)		.73	

^a^Includes only participants with data at both enrollment and follow-up visits.

^b^Linear generalized estimating equations (coefficient interpretable as an increase in the score from enrollment to follow-up).

^c^ART: antiretroviral therapy.

^d^IMB: information, motivation, and behavioral skills.

^e^Maximum score of 100.

^f^Patient Health Questionnaire-9 (PHQ-9) score; maximum score of 27.

### Pre-Post Change in Psychosocial Outcomes

We found no significant difference in depressive symptoms and social support scores (Table 5). However, we observed an increase in the median stigma score from 8.3 (out of 100; IQR 0.0-16.7) to 16.7 (IQR 0.0-33.3), an estimated 10.3-point increase (95% CI 3.7-16.6; *P*=.001). In particular, the subscale focusing on internalized stigma showed an increase of 10.1 (95% CI 2.3-17.9; *P*=.01). Additionally, we observed a decrease in the resilience score from 75.0 (out of 100; IQR 65.0-85.0) to 70.0 (IQR 55.0-80.0; *P*<.001).

### Association Between Messaging Level and Outcomes

We investigated whether participant engagement in the intervention was associated with a pre-post change in outcomes. Table 6 presents the associations of 3 measures of engagement (leaving the group at any time, total number of messages sent, and number of days a participant was active) with outcomes of interest, adjusting for baseline outcome levels. Ever leaving the group was associated with an 11.9% lower ART adherence score (IQR 2.59%-22.20%) and an 11.37% higher enacted stigma score (IQR 1.33%-21.41%). There was a trend for a positive association between the number of active days and the resilience score: 10 additional active days were associated with a 2.37% increase in the resilience score (IQR −0.02 to 4.76). No other outcomes were significantly associated with engagement.

**Table 6:** Association between participant engagement with the intervention and HIV and psychosocial outcomes at follow-up.

Outcome^a^			Left the group at any time				Total message number				Number of days active		
			Coefficient (95% CI)		*P* value		Coefficient (95% CI)^b^		*P* value		Coefficient (95% CI)^c^		*P* value
**ART^d^ IMB^e^ score^f^**			2.02 (−5.10 to 9.14)		.58		0.06 (−0.33 to 0.45)		.77		−0.40 (−1.58 to 0.78)		.51
	ART information	−1.60 (−-10.2 to 7.00)		.72		0.30 (−0.19 to 0.79)		.24		0.46 (−1.09 to 2.01)		.56	
	ART motivation	1.68 (−10.05 to 13.41)		.78		−0.26 (−0.94 to 0.73)		.46		0.94 (−3.01 to 1.13)		.38	
	ART behavioral skills	−0.73 (−11.07 to 9.62)		.89		−0.06 (−0.65 to 0.54)		.85		1.06 (−2.86 to 0.73)		.25	
ART adherence^f^			−11.90 (−22.20 to −1.59)		.02		0.21 (−0.41 to 0.83)		.51		0.37 (−1.57 to 2.31)		.71
Depression score^g^			−1.36 (−3.58 to 0.86)		.23		0.02 (−0.11 to 0.14)		.81		0.09 (−0.30 to 0.49)		.64
**Stigma score^f^**			5.67 (−7.98 to 19.31)		.42		0.16 (−0.62 to 0.95)		.69		0.74 (−1.61 to 3.09)		.54
	Anticipated stigma	7.02 (−21.03 to 35.07)		.62		0.47 (−0.98 to 1.92)		.52		1.83 (−2.64 to 6.29)		.42	
	Enacted stigma	11.37 (1.33 to 21.41)		.03		−0.18 (−0.79 to 0.43)		.57		−0.41 (−2.26 to 1.43)		.66	
	Internalized stigma	2.03 (−13.60 to 17.67)		.80		0.08 (−0.82 to 0.99)		.86		0.38 (−2.41 to 3.17)		.79	
Resilience score^f^			−9.02 (−23.25 to 5.21)		.21		0.69 (−0.06 to 1.45)		.07		2.37 (−0.02 to 4.76)		.05
Social support score^f^			−3.12 (−13.27 to 7.03)		.55		−0.13 (−0.68 to 0.43)		.65		−0.22 (−2.05 to 1.61)		.81

^a^Analyses evaluate the association between engagement measures as predictors and HIV-related outcomes as outcomes. All analyses exclude 3 participants who did not message on their own devices but shared phones with 3 other participants.

^b^Increase in the outcome score associated with sending 20 additional messages over the course of the intervention. Adjusted for the baseline outcome score.

^c^Increase in the outcome score associated with being active on 10 additional days over the course of the intervention. Adjusted for the baseline outcome score.

^d^ART: antiretroviral therapy.

^e^IMB: information, motivation, and behavioral skills.

^f^Maximum score of 100.

^g^Maximum score of 27.

## Discussion

### Principal Findings

In this pilot study, we evaluated the uptake, utility, and preliminary effect of a novel WhatsApp-based peer group intervention for youth living with HIV in Nairobi, Kenya. We found that participants expressed high satisfaction with the intervention. All eligible youth chose to enroll in the study, and the vast majority reported that the intervention was helpful for learning new information, gaining emotional support, and promoting ART adherence. The levels and patterns of participant engagement varied considerably between participants. Most participants messaged the group at some point, but typically on a minority of days during the intervention. Messaging was higher during school holidays in the group of ≥18-year-old individuals and declined over time. On comparing baseline and follow-up data among participants, we found a significant improvement in participants’ ART-related information and behavioral skills, which are indicators associated with ART adherence behavior [26], consistent with our hypothesis that a peer support intervention could support participants’ engagement in HIV care. However, despite participants’ high satisfaction and perceived benefit, we found no improvement in ART adherence or social support. Additionally, we found a significant increase in stigma scores and a decrease in resilience scores, which were unexpected. We detected a positive association between the level of messaging during the study and the resilience score, but no significant association between messaging and other outcomes. Our pilot study also shed light on the feasibility and challenges of recruiting and retaining youth living with HIV in a social media group intervention. In-person outreach by study or clinic staff was the most effective mode of recruitment, while snowball sampling had a low yield. Once enrolled, it was common for participants to change phone numbers or leave the groups and request to be added back, which may present implementation challenges at a larger scale.

### Comparison With Prior Work

Previous studies evaluating digital health interventions for ART adherence and virologic suppression have mostly focused on SMS text messaging connecting adults with health care workers, and have yielded mixed evidence [12,13,27-32]. Few studies have evaluated digital health interventions among youth living with HIV [15]. A recent randomized controlled trial (RCT) reported improved ART adherence in Guatemalan youth receiving informational SMS text messages [33], while other studies have not found significant effects of SMS text message interventions in youth [30,34,35]. As global access to smartphones and social media has expanded, the use of technology to facilitate peer-to-peer interventions for youth living with HIV has increased. However, studies of virtual peer group interventions have been small, and few have been robust randomized evaluations [14]. Our findings of improved ART information and behavioral skills are consistent with the findings of Dulli et al of improved ART knowledge in an RCT of a Facebook intervention among Nigerian youth living with HIV [36]. Despite high satisfaction and improved ART information and behavioral skills, we did not detect improvement in self-reported ART adherence or social support. This may be explained by our small sample size and short follow-up time. A previous study of a web-based peer group intervention also reported increased ART adherence intention but no significant improvement in self-reported ART adherence after 3 months [37]. Additionally, owing to our nonrandomized design, we could not determine how these measures would have changed in the absence of the intervention.

Our observation of increased stigma and decreased resilience scores was unexpected and requires further study. It is possible that hearing about peers’ lived experiences in the Vijana-SMART group led to heightened awareness of stigma and the challenges of living with HIV, or that discussion of stigma in the group increased participants’ ability to identify their experiences as stigma. Internalizing these ideas may also have led to decreased resilience scores. During in-depth interviews at the study exit, participants reported enhanced support as a result of participation and did not report any adverse impact [25]. Our finding of increased stigma is consistent with a recent pilot study of a similar WhatsApp group intervention in youth living with HIV in Western Kenya; the prior study did not report resilience scores [38]. Future group interventions could track the duration or evolution of stigma and resilience, target stigma and resilience more directly, and potentially identify participants at heightened risk for these effects.

To shed light on potential intervention mechanisms, we analyzed the association of participants’ messaging levels with baseline characteristics and behavioral outcomes. mHealth intervention usage data (paradata) can explain mechanisms and optimize intervention targeting, but they have been understudied [39]. We hypothesized that participants who engaged more with the Vijana-SMART group would exhibit greater improvement in behavioral outcomes. Our findings did not support this hypothesis. It is possible that the relationship between engagement and behavioral outcomes is more complex than a linear association between messaging frequency and outcome score. For example, in a study of an online weight gain prevention intervention, Graham et al found that the intervention effect was associated nonlinearly with specific modes of engagement, and that these associations were not consistent across different strata of participants [40]. It is also possible that sending messages is not a meaningful indicator of a participant’s engagement, and perhaps simply reading intervention messages (a behavior not captured in our paradata) can modify behavioral outcomes. We found modest associations of leaving the group with ART adherence and enacted stigma, as well as messaging frequency with resilience scores, although these correlations cannot be interpreted as causation, and the analysis was not corrected for multiple comparisons. Nevertheless, the associations are intriguing considering the overall increase in stigma and decline in resilience scores from enrollment to follow-up. Together, these findings suggest that the unexpected changes in stigma and resilience in the cohort as a whole may have been driven by participants who were less engaged in the intervention. More in-depth analysis of patterns of engagement with Vijana-SMART in a larger cohort may provide valuable insights about its effect.

### Limitations

Our study adds to the small but growing literature on the use of social media group interventions for youth living with HIV [14,41]. The strengths of our study include participatory and theoretically grounded intervention development. We were able to implement the intervention without having to supply phones or airtime, providing information about real-world feasibility. Our analysis of participant engagement and behavioral outcomes sheds light on the intervention’s mechanism. The study is limited by its nonrandomized design, small sample size, and short follow-up. Loss to follow-up was moderate (16%), so our complete case sample may overrepresent participants who were better engaged in care. Our intervention messages were based on social support theory and combined light-touch prosocial messages with unstructured discussions with peers and the facilitator to support the development of behavioral skills. The intention of this design was to elevate peer voices, which are known to be important influences on youth behavior, but it may involve limited standardization of behavioral skills development. Our messages were grounded in social support theory, whereas our assessment was grounded in the IMB theory. It is possible that closer alignment between intervention elements and evaluation tools would have yielded more informative data. Additionally, the use of WhatsApp without supplying devices relies on youth’s access to smartphones. In formative work, we found that 52% of youth living with HIV at 3 health care facilities in Nairobi had access to smartphones [42]. While smartphone access is expected to continue expanding, the intervention is not currently universally accessible and may exclude the most marginalized youth.

### Conclusions

This study presents a novel digital intervention offering youth living with HIV information and support from peers and a trained facilitator. The intervention design was grounded in guidance for youth living with HIV, leading to an acceptable and feasible intervention with promising initial findings. The findings may be especially relevant in the context of COVID-19–related disruption of in-person health care. Future research is needed to deepen the interpretation of these findings. In particular, a larger-scale randomized evaluation of Vijana-SMART with long-term follow-up will support more robust testing of the intervention’s efficacy. More detailed assessment of stigma and resilience is also needed, including longitudinal quantitative assessment and qualitative exploration of these constructs. Finally, the collection of more detailed paradata, such as reading of messages in addition to sending of messages, is needed to better understand the relationship between intervention engagement and outcomes.

## Appendix

Multimedia Appendix 1 Intervention messages of the Vijana-SMART intervention for youth living with HIV in Kenya.

## Abbreviations

ART: antiretroviral therapy
GEE: generalized estimating equation
IMB: information, motivation, and behavioral skills
mHealth: mobile health
PHQ-9: Patient Health Questionnaire-9
RCT: randomized controlled trial

## Data Availability

The data sets analyzed during this study are available on GitHub [43].
