# Supplementary material for: Facilitated WhatsApp Support Groups for Youth Living With HIV in Nairobi, Kenya: Single-Arm Pilot Intervention Study
Source: JMIR Form Res. 2023 Nov 13;7:e49174. doi: 10.2196/49174 (PMC10682925; doi:10.2196/49174)
Supplement: Multimedia Appendix 1 [file formative_v7i1e49174_app1.docx]

**Multimedia Appendix 1.** Intervention messages of the Vijana-SMART intervention for youth living with HIV in Kenya.

| Week | Topic | Scheduled message |
| --- | --- | --- |
| 0 | Greetings | Hi everyone and welcome to the Vijana-SMART group! 👋🏾 We are excited to have you join us! 😃 |
| 0 | Housekeeping | With this group, we would like to support each other in staying healthy and supported. If you are having problems or questions about your health, please share them with the group, or inbox me individually. We are stronger together 🤝 |
| 0 | In-person launch meeting | You are invited to XXX clinic for a fun day to learn about the group next week on April 6 at XXXtime. This meeting is optional, please let us know if this date and time will work for you. Please bring your WhatsApp phone with you. We are excited to meet you! |
| 0 | Building connection | Let's get to know each other in this group. What's your favourite way to spend a day off from work or school? What music are you enjoying lately? 🎶🕺🏿 XXX [*facilitator can share his responses to get the group started]* XXX |
| 1 | Housekeeping | Hi friends! This is our weekly SMART message! It was nice to see some of you this week at our meeting. Here is quick a reminder of the agreements we made at our launch meeting about how to use this group: ✅[share agreements from in-person meeting] ✅ [agreement] ✅… |
| 1 | Housekeeping | With this group, we would like to support each other in staying healthy and supported. If you are having problems or questions about your health, please share them with the group, or inbox me individually. We are stronger together 🤝 |
| 1 | Building connection | How are we all doing this week? What are you most looking forward to about this group? |
| 2 | Companionship | How are we all this week? What has happened in the last week? Anything fun or exciting that we can celebrate together? 😂🥳 Or something hard that you want support with? 😒😢 |
| 3 | Information/instrumental: ART^a^ adherence | Hello! This is our weekly SMART message! Having trouble taking medication 💊💊daily? Let us share our experiences. |
| 4 | Emotional support/companionship | Hello! This is our weekly SMART message! Young people are in a time of change in our lives! We all have hopes and dreams for what we want as we get older. 💭🔮What do you hope to do in the next few years? |
| 5 | Instrumental: ART reminders | Hello! This is our weekly SMART message! Guys let's remind each other 💊. Tell us next time you take! |
| 6 | Information/instrumental support: food | Hello! This is our weekly SMART message! it’s very important that we have a balanced diet – especially when we are young and more so when we are taking medication. 🍲 How are we ensuring that we are achieving our nutritional goals? |
| 6 | Information/instrumental support: food | Here's an idea for a balanced meal: half a loaf of bread is Ksh. 20, 2 eggs Ksh. 20, a banana Ksh. 5, tomato Ksh. 5. Does anyone have other tips on easy balanced meals to make? |
| 7 | Emotional support: general encouragement | Hello! This is our weekly SMART message! Remember a problem shared is a problem half solved. We are in this together! 💌🙂 If there is a problem/questions please share or inbox me. |
| 8 | Information: side effects | Hello! This is our weekly SMART message! Some people experience temporary uncomfortable changes in their body due to taking medication. 🥴🤢😴 Has anyone ever experienced this? Please share what you did to cope with this? |
| 9 | Emotional support: encouragement | Hello! This is our weekly SMART message! How is everyone doing this week? Am sure you guys are having experiences that others can relate to and advise on. 🤔💃🏿 |
| 10 | Emotional support: disclosure | Hello! This is your weekly SMART message! You can only walk with people who know where you're going. To walk with you people need to know your situation. 👣👣 Many of us have shared with others about their health. Please share with us about your experiences?  https://www.avert.org/hubs/young-voices-africa/talking-about-hiv |
| 12 | Information: positive prevention | Hello! This is our weekly SMART message! Have you heard about prevention with positives? We could be in relationships with people who are not with the same status. How can we protect our loved ones? 🥰 |
| 13 | Companionship | Hello! This is our weekly SMART message! How are we all this week? Does anyone want to share anything that happened in the last week? It can be something for us to laugh or celebrate together, or something hard we can support each other about. We are together! 🤝🤝🤝 |
| 14 | Emotional support: stigma | Hello! This is our weekly SMART message! Sometimes, people with long-term illness experience stigma, meaning other people do not value them and view them negatively because something is different about them. This is hurtful and unfair. We sometimes take in these ideas and even feel negative judgements towards ourselves (self-stigma). 😔😧😢 Have any of you experienced this? What has helped you overcome this?💡 |
| 15 | Information/emotional: mental health | Hello! This is our weekly SMART message! Sometimes life is stressful and we feel down and discouraged. 😞😨😣. We all deserve to be healthy and there are things we do to feel better in these times! Please share how you cope with such situations. |
| 16 | Instrumental: relationship skills | Hello! This is our weekly SMART message! As young people, we may be exploring new relationships and experiences. 🥰🥰 This can be exciting! But can also put us in new situations we feel unsure about.🤔🤨 Has anyone had any situations they were unsure about in relationships with other people? What has helped you navigate them to stay true to your life goals? |
| 17 | Emotional support: encouragement | Hello! This is our weekly SMART message! Wow 3 months has really passed quickly!!⏳ 😮Thanks to everyone for all your messages and support. This is really appreciated 😄 We are looking forward to another 3 gr8 months. Are there any questions you have or topics you would like us to discuss during the rest of our time together? Please message me or the whole group to let us know what would help you. 💪🏿 |
| 18 | Information: substance use | Hello! This is our weekly SMART message! Remember we need to take extra care of our bodies. This includes avoiding drugs, including alcohol🍺 and tobacco🚬. Even though we may experience peer pressure, they alter how we make decisions and interact with our medication. How do you keep your smoking and drinking low when others around you are doing it? |
| 19 | Companionship | Hello! This is our weekly SMART message! What has been the high point 🌟 and low point ☹️ of your week ? We are together for good times and bad! |
| 20 | Instrumental support: transition | Hello! This is your weekly SMART message! As we get older, we become more independent. We also start to manage our medical care independently. What does managing your care independently mean to you?💊🚌📉📝 What skills do you think you need to learn before you are ready? Others in the group may have experiences that can help you learn and prepare.🤔 |
| 21 | Instrumental support: transition | Hello! This is your weekly SMART message! As we prepare for managing our care independently, it is important to feel comfortable asking questions in our clinic appointments - just like we ask question in this group! Do you feel comfortable asking the nurse questions about your test results, medicine, side effects? ❓🤷🏾‍♀️⁉️ |
| 22 | Insufficient food as a barrier to adherence | Hello! This is your weekly SMART message! Some medications need to be taken with food - but sometimes we lack food. Even when we don't have much money, we can get enough food to take our daily dose. It can just be a small snack like a banana or some roasted maize. Here's an idea for a balanced meal: half a loaf of bread is Ksh. 20, 2 eggs Ksh. 20, a banana Ksh. 5, tomato Ksh. 5. Does anyone have other tips on getting enough food before taking medications? 🍌🌽🥚🍞🍅 |
| 23 | Housekeeping | Vijana-SMART family, we are starting the last month of our 6-month study group!! Over the next few weeks, I will recap some topics and resources we have discussed before. Please ask me any other questions you have - to the group or direct message. |
| 23 | Informational support: transition | Hello! This is our weekly SMART message! A few weeks ago we talked of transition to adult care and some of us had questions about it. In the adult clinic you will be seen and pick up your refills without your caregiver. If you have a side effect or question you will talk to the nurse yourself. Can anyone share what it's like when you are seen with the adults? We can share our experiences to make this less confusing! 😵 |
| 24 | Informational support: Disclosure & U=U | Hello! This is our weekly SMART message! Over the last few weeks, we have discussed that undetectable means untransmissible (U=U!). Some of you have asked. "Do I need to disclose to my sex partner if I am undetectable?" Here is my answer. Having undetectable VL for at least six months AND continuing to stay on medication means you are not putting your partner at risk. BUT consider the advantages of disclosing. ⚖️ Disclosing makes it possible for your partner to support you. Also a partner may become upset if they learn about your status after sex and it can cause unnecessary conflict even when there is no risk of transmission. Also, it’s extremely important that this is ONLY true if you stay undetectable - if you are not sure of your VL, you may be putting your partner at risk. Do you have any questions about this? |
| 25 | Informational support: disclosure - APS | Hello! This is our weekly SMART message! A few weeks ago we discussed APNS (assisted partner notification services). This is a service available at many public facilities where a trained counselor advises on disclosure options. This can mean making a plan with you about how to disclose, or the counselor can contact the partner and encourage them to be tested - without mentioning your name. Many people have found APNS helpful. Have any of you used it? In our area, here are some facilities where you can get this service: XXX |
| 26 | Opportunistic infections | Hello! This is our weekly SMART message! We are often told that we are at risk of opportunistic infections, but what are some signs of infection? If you experience fever, coughing, diarrhea, vomiting, or a rash on your skin or in your mouth, go to the clinic.🥴🤮🤒 Have you had any symptoms that worried you? |
| 26 | Housekeeping | After October, you can continue to message each other but I will no longer be the facilitator. At the end of the study in October, we will have a group gathering for all of you at Kayole II clinic, as you suggested! We were thinking of having this on the last week of October - let us debate when is the best time for everyone. We will also call you to arrange individual study exit visits to hear your experience of the group. |
| 27 | Information: substance use & mental health | Hello! This is our weekly SMART message! Sometimes life is hard and we feel down, discouraged or bored. 😞😨😣 Sometimes we try to feel better by taking alcohol or drugs to make us feel different. This can feel like it helps - but it actually just makes us feel worse in the long-term and hurts our health. Instead, here are some ideas that can help your mood when you are down: meet up with a friend 👫, take a walk or run outside 🏃🏿‍♀️, listen to your favourite music 🕺🏿- or message your Vijana-SMART family 😍! Any other ideas? What do you do when you feel down? |
| 28 | Safer sex, prevention | Hello! This is our weekly SMART message! As we get older and have sexual relationships, it's important to protect ourselves and our partners from STIs and unwanted pregnancy. Barrier methods like condoms prevent STIs and pregnancy. There are many other methods to prevent pregnancy (they don't prevent STIs) https://www.avert.org/sites/default/files/Options%20for%20contraception%20-%20Worksheet.pdf. Also, for HIV, if we are not undetectable, our negative partner can take PrEP to protect themselves. https://twitter.com/nimejiprep?lang=en |
| 29 | Goodbye | Thank you all for your participation in our group over the last 6 months!!! 🙏🏿It's been a pleasure to be here with you and support each other!! Our time together is ending now.👋🏾 Please remember to come to your study exit visit and tell us how about your time in the group so we can improve it for future vijana! |
